# The Evolving Role of Critical Access Hospitals in Rural Physician Training

**DOI:** 10.1001/jamahealthforum.2025.4742

**Published:** 2025-10-31

**Authors:** Lori Rodefeld, Mukesh Adhikari, Catherine (Kasia) Horger, John Boll, Emily M. Hawes

**Affiliations:** 1 Wisconsin Collaborative for Rural Graduate Medical Education, Sauk City; 2 Cecil G. Sheps Center for Health Services Research, University of North Carolina, Chapel Hill; 3 Department of Health Policy and Management, Gillings School of Global Public Health, University of North Carolina, Chapel Hill; 4 Duke Health Integrated Practice, Durham, North Carolina; 5 University of Pittsburg Medical Center, Williamsport, Pennsylvania; 6 Department of Family Medicine, School of Medicine, University of North Carolina, Chapel Hill

## Abstract

This cross-sectional study examines the prevalence of critical access hospital–based training and compares these sites with other rural teaching hospitals to understand potential for expansion.

## Introduction

The 1364 critical access hospitals (CAHs) across the US—hospitals with 25 or fewer beds often located in rural locations delivering essential health care services—represent an emerging option to increase rural graduate medical education (GME).^1^ Between 2000 and 2009, only 3 to 11 CAHs were reported as hosting residents.^2^

Recent policy reforms enhancing GME flexibility have created growth opportunities, including streamlined accreditation for Rural Track Programs (RTPs) and changes in Medicare reimbursement for urban hospitals partnering with CAHs.^3^ Federal initiatives such as the Rural Residency Planning and Development (RRPD) program support GME expansion in CAHs through startup funding and technical assistance.^4^ This cross-sectional study examines the prevalence of CAH-based training and compares these sites with other rural teaching hospitals to understand potential for expansion.

## Methods

We analyzed 2023 to 2024 data from the Accreditation Council for Graduate Medical Education (ACGME) Data System, including program codes, site addresses, and months at each site. A list of CAHs was obtained from 2023 hospital data.^1^ We geocoded sites and CAHs using ArcGIS Pro 3.2.2 (Esri), creating a 8-km (5-mile) buffer around each CAH, and performed a spatial join to identify overlapping sites. Matches were validated by cross-referencing names and addresses. As over 95% of CAHs are rural, we compared them with rural teaching hospitals, using 2022 Medicare Cost reports^5^ and 2022 County Health rankings data.^6^ We received an exemption determination from the University of North Carolina institutional review board because the study did not involve human participants and followed the STROBE reporting guideline.

## Results

Table 1 describes characteristics of 80 programs with CAH-participating sites, representing a median of 6.5% (IQR, 2.8%-53.5%) training time in CAHs. Family medicine, internal medicine, and surgery programs train most frequently in CAHs. Twenty-three programs offer at least 50% training in CAHs, while 57 programs offer CAH rotations. Fifteen new programs with training in CAHs attained accreditation during the study period. Examples include Oregon Health & Science University Three Sisters RTP—a CAH partnered with a Federally Qualified Health Center and Tribal Clinic—and University of Pittsburgh Medical Center’s family medicine RTP.^4^ Table 2 compares 93 CAH training sites with 171 rural non-CAH teaching hospitals. CAHs serve higher percentages of older adults (20.3% vs 19.1%), Hispanic individuals (5.7% vs 3.5%), and uninsured children (5.8% vs 4.9%) compared with rural teaching hospitals and have comparable primary care physician to population ratios (5.7 vs 6.3). Of RRPD programs, 31 of the sites are CAHs.^4^

**Table 1.** Characteristics of Residency Programs Training Residents in CAHs

Characteristic	No. (%) (N = 80)
Specialty	
Family medicine	55 (68.8)
Internal medicine	9 (11.3)
Surgery	9 (11.3)
Obstetrics and gynecology	4 (5.0)
Psychiatry	2 (2.5)
Pediatrics	1 (1.3)
Training in CAH, median (IQR), %	6.5 (2.8-53.5)
Program with ≥50% training at CAH	
Yes	23 (28.8)
No	57 (71.3)

Abbreviation: CAH, critical access hospital.

**Table 2.** Characteristics of CAH Training Sites Compared With Other Rural Teaching Hospitals

Characteristic	Median (IQR)		*P* value^a^
	CAH-training sites (n = 93)^b^	Rural teaching hospitals (n = 171)	
Total margin, %	10.9 (5.0-16.7)	8.9 (2.7-17.6)	.48
Operative margin, %	0.1 (0.0-0.1)	0.1 (0.0-0.1)	.42
Acute beds, No.	25.0 (23.0-25.0)	95.0 (50.0-160.0)	<.001
Income below the federal poverty level, %	12.6 (9.6-15.3)	13.9 (10.8-18.1)	.007
Non-Hispanic Black population, %	0.8 (0.5-1.9)	2.6 (1.2-8.7)	<.001
Hispanic population, %	5.7 (3.2-10.7)	3.5 (2.1-7.0)	<.001
Population aged ≥65 y, %	20.3 (17.5-23.7)	19.1 (17.3-20.9)	.006
Population aged <18 y, %	22.0 (19.0-23.6)	21.6 (19.9-23.3)	.94
Uninsured adults, %	12.5 (8.8-17.5)	11.1 (8.8-15.2)	.22
Uninsured children, %	5.8 (4.2-8.1)	4.9 (4.0-6.3)	.01
Population with fair or poor health, %	19.1 (16.6-21.9)	20.7 (18.1-24.4)	.004
Age-adjusted death rate per 100 000 population	371.7 (313.5-435.2)	432.3 (364.7-534.9)	<.001
Primary care physicians per 10 000 population	5.7 (3.9-8.1)	6.3 (4.6-8.4)	.11
Mental health clinician rate per 10 000 population	17.1 (9.2-27.9)	21.2 (12.7-35.3)	.005

Abbreviation: CAH, critical access hospital.

^a^ *P* < .05 was considered statistically significant.

^b^ Three of 96 CAHs did not merge with 2022 Medicare Cost Report Data.

## Discussion

CAH participation in GME has substantially increased since the early 2000s, with nearly 100 CAHs engaged in training. Despite lower bed size compared with other rural teaching hospitals, CAHs offer similar financial and operative characteristics. Comparable primary care physician to population ratios at CAHs and rural teaching hospitals suggest a similar faculty supply for GME training, albeit lower than metropolitan areas.

Expansion of CAH participation in GME may stem from policy reform, regulatory changes, and startup funding. Financing for training in CAHs has facilitated growth as Medicare reimburses urban hospitals offering rotations by treating the CAH as a nonprovider site through a funds flow agreement.

Study limitations include potential underestimation of CAH training, given that most resident time is claimed on urban hospital Medicare reports and there is variability in program reporting of rotations to ACGME. Although financial regulations have created sustainable pathways, CAHs without urban partners cannot receive full reimbursement and are paid on a reasonable cost basis. Extending nonprovider payment models to other sites, such as tribal facilities, could bolster rural GME.

CAH participation in GME has increased. With only 7.0% of CAHs (96 of 1364) training residents, there is potential to expand training through CAHs, which often serve isolated areas lacking GME. Given the return on investment of the rural GME to the physician workforce pathway, the growing integration of CAHs into GME underscores how policies that leverage funding mechanisms through partnerships can broaden rural training.

## Notes

Supplement.
Data Sharing Statement
