# Supplementary material for: The Evolving Role of Critical Access Hospitals in Rural Physician Training
Source: JAMA Health Forum. 2025 Oct 31;6(10):e254742. doi: 10.1001/jamahealthforum.2025.4742 (PMC12579338; doi:10.1001/jamahealthforum.2025.4742)
Supplement: Supplement. — Data Sharing Statement [file jamahealthforum-e254742-s001.pdf]

Data Sharing Statement

Rodefeld. The Evolving Role of Critical Access Hospitals in Rural Physician Training. *JAMA Health Forum*. Published October 31, 2025. doi:10.1001/jamahealthforum.2025.4742

Data
Data available: Yes
Data types: Data (not involving human participants), Data dictionary
How to access data: Data may be made available upon reasonable request with research proposal
When available: With publication

Supporting Documents
Document types: None

Additional Information
Who can access the data: Researchers whose proposed use of the data has been approved
Types of analyses: Health Services Research
Mechanisms of data availability: After approval of a proposal
Any additional restrictions: none
